# 20(S)-Rg3 blocked epithelial-mesenchymal transition through DNMT3A/miR-145/FSCN1 in ovarian cancer

**DOI:** 10.18632/oncotarget.18482

**Published:** 2017-06-15

**Authors:** Jie Li, Jiaojiao Lu, Zhongxue Ye, Xi Han, Xia Zheng, Huilian Hou, Wei Chen, Xu Li, Le Zhao

**Affiliations:** ^1^ Center for Translational Medicine, The First Affiliated Hospital of Xi’an Jiaotong University, Xi’an, China; ^2^ Key Laboratory for Tumor Precision Medicine of Shaanxi Province, The First Affiliated Hospital of Xi’an Jiaotong University, Xi’an, China; ^3^ Department of Gynecology and Obstetrics, The First Affiliated Hospital of Xi’an Jiaotong University, Xi’an, China; ^4^ Department of Gynecology, Ningbo No. 2 Hospital, Ningbo, China; ^5^ Department of Pathology, The First Affiliated Hospital of Xi’an Jiaotong University, Xi’an, China; ^6^ Center for Laboratory Medicine, The First Affiliated Hospital of Xi’an Jiaotong University, Xi’an, China

**Keywords:** ovarian cancer, ginsenoside, microRNA, methylation, epithelial-mesenchymal transition

## Abstract

Epithelial-mesenchymal transition (EMT) is one of the key mechanisms mediating cancer progression. MicroRNAs (miRs) are essential regulators of gene expression by suppressing translation or causing degradation of target mRNA. Growing evidence illustrates the crucial roles of miRs dysregulation in cancer development and progression. Here, we have found for the first time that the ginsenoside 20(S)-Rg3, a pharmacologically active component of Panax ginseng, potently increases miR-145 expression by downregulating methyltransferase DNMT3A to attenuate the hypermethylation of the promoter region in the miR-145 precursor gene. Restoration of DNMT3A reverses the inhibitory effect of 20(S)-Rg3 on EMT. FSCN1 is verified as the target of miR-145 to suppress EMT in human ovarian cancer cells. The results from nude mouse xenograft models further demonstrate the suppressive effect of miR-145 on malignant progression of ovarian cancer. Taken together, our results show that 20(S)-Rg3 blocks EMT by targeting DNMT3A/miR-145/FSCN1 pathway in ovarian cancer cells, highlighting the potentiality of 20(S)-Rg3 to be used as a therapeutic agent for ovarian cancer.

## INTRODUCTION

Ovarian cancer is the most lethal gynecological tumor, existing predominantly in the form of epithelial ovarian cancer (EOC)[1, 2]. The prognosis of EOC patients remains poor, and cancer metastasis and recurrence are the major causes of death. Emerging evidence suggests that the epithelial-mesenchymal transition (EMT), the conversation of epithelial cells to fibroblast-like cells mainly characterized by loss of epithelial molecules, acquisition of mesenchymal markers, enhancement of cell mobility and invasion, plays a crucial role in the progression of EOC by increasing cancer cell invasion and metastasis [3]. Thus, targeting EMT process for novel anticancer drug discovery is highly significant for the clinical benefits of ovarian cancer patients.

Ginsenosides are the pharmacologically active components of Panax ginseng that has long been utilized as a traditional Chinese medicine for officinal or recuperative purposes [4–6]. To date, more than 100 ginsenoside compounds have been identified [7], among which Rg3 is one of the bioactive extracts with anti-tumor effect [8, 9]. Ginsenoside Rg3 has two stereoisomers 20(R)-Rg3 and 20(S)-Rg3, which differ in the orientation of the hydroxyl (OH) group on carbon-20 [5]. We have found that 20(S)-Rg3, rather than 20(R)-Rg3, has robustly blocked hypoxia-induced EMT in ovarian cancer.

Many EMT players such as E-cadherin transcription repressors including snail, zeb and Twist, and microRNAs have been identified so far. MicroRNAs are small non-coding RNAs that modulate gene expression at the post-transcriptional level through complementary binding to 3′UTR of target mRNAs [10]. Some microRNAs including miR-145, miR-200, and miR-29b, to name a few, have been suggested as EMT repressors in various types of cancer [11–14]. miR-145 has been downregulated in many cancers [15–19], functioning as tumor suppressor to inhibit tumor cell growth and survival, induce cell apoptosis and cell cycle arrest, and attenuate tumor cell migration and invasion via targeting various molecules [16–18, 20–23]. Reduction of miR level in cancers can be associated with aberrant epigenetic regulation such as DNA hypermethylation [24]. DNMT3A and DNMT3B are two major de novo DNA methyltransferases in mammals, while DNMT1 is in charge of maintenance of DNA methylation [25]. DNMT3A and DNMT3B have been reported upregulated in ovarian cancers [26]. Both of them have been able to hypermethylate the promoter region in microRNA precursor genes and thus inversely regulate microRNA transcription.

In the present study, we discovered that 20(S)-Rg3 enhanced miR-145 expression by downmodulating DNMT3A to attenuate the methylation level in the promoter region of miR-145 precursor gene. The promotion of miR-145 by 20(S)-Rg3 directly targeted FSCN1 to reverse EMT *in vitro* and *vivo*. These results not only uncovered the novel anti-cancer mechanism of 20(S)-Rg3, but also revealed the regulatory pathway for miR-145 expression.

## RESULTS

### 20(S)-Rg3 reversed EMT via upregulating miR-145

**Figure 1:**
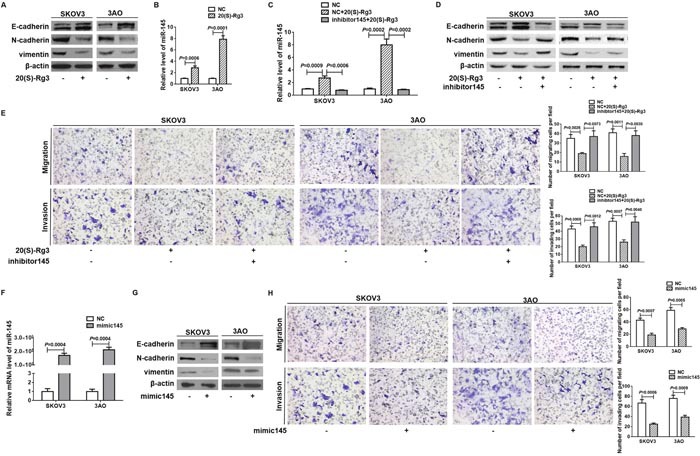
20(S)-Rg3 reversed EMT via upregulating miR-145 **(A)** Western blot analysis showed that epithelial marker E-cadherin was up-regulated while mesenchymal markers N-cadherin and vimentin were down-regulated at protein level in cells treated by 20(S)-Rg3. **(B)** qRT-PCR showed that 20(S)-Rg3 increased miR-145 level in SKOV3 and 3AO cells. **(C)** qRT-PCR showed that inhibition of miR-145 antagonized 20(S)-Rg3-triggered increase of miR-145. **(D)** Western blot analysis indicated that E-cadherin increase, N-cadherin and vimentin decrease caused by 20(S)-Rg3 were reversed by concomitant miR-145 downregulation. **(E)** *In vitro* migration and invasion assay showed that transfection of miR-145 inhibitor reversed the inhibitory effect of 20(S)-Rg3 on cell motility and invasion (200×). **(F)** qRT-PCR showed that transfection of miR-145 mimic significantly increased miR-145 level in SKOV3 and 3AO cells. **(G)** Western blot assays showed that the expression of E-cadherin was increased and N-cadherin and vimentin were decreased in SKOV3 and 3AO cells transfected with mimic145. **(H)** *In vitro* migration and invasion assays showed that overexpression of miR-145 in SKOV3 and 3AO cells reduced the cell invading capacity (200×). All experiments were carried out in triplicate and the results were presented as means ± SE. t-test.

In 20(S)-Rg3-treated SKOV3 and 3AO cells, epithelial marker E-cadherin was up-regulated, while mesenchymal markers N-cadherin and vimentin were down-regulated (Figure 1A), indicating that 20(S)-Rg3 reversed EMT in SKOV3 and 3AO ovarian cancer cells. Meanwhile, 20(S)-Rg3 caused E-cadherin increase and N-cadherin and vimentin decrease in A2780 ovarian cancer cells and HEC-1-B endometrial cancer cells (Supplementary Figure 1A). Since miR-145 was reported downregulated in ovarian cancer cells and involved in EMT in many types of cancer, we explored the effect of 20(S)-Rg3 on miR-145. In both SKOV3 and 3AO cells, 20(S)-Rg3 stimulated miR-145 expression (Figure 1B). Transfection of miR-145 inhibitor (Supplementary Figure 2) into 20(S)-Rg3-treated cells (Figure 1C) reversed 20(S)-Rg3-rendered E-cadherin upregulation and N-cadherin and vimentin downregulation (Figure 1D, Supplementary Figure 3). Inhibition of miR-145 also blocked the inhibitory effect of 20(S)-Rg3 on the migration and invasion of both SKOV3 and 3AO cells (Figure 1E). We next overexpressed miR-145 to examine its effect on EMT of ovarian cancer cells. miR-145 overexpression (Figure 1F) increased E-cadherin while decreased N-cadherin and vimentin (Figure 1G). Meanwhile, overexpression of miR-145 largely inhibited migration and invasion of both SKOV3 and 3AO cells (Figure 1H). These results showed that 20(S)-Rg3 upregulated miR-145 to reverse EMT in ovarian cancer cells.

### 20(S)-Rg3 upregulated miR-145 via suppressing DNMT3A to demethylate pre-miR-145 gene

**Figure 2:**
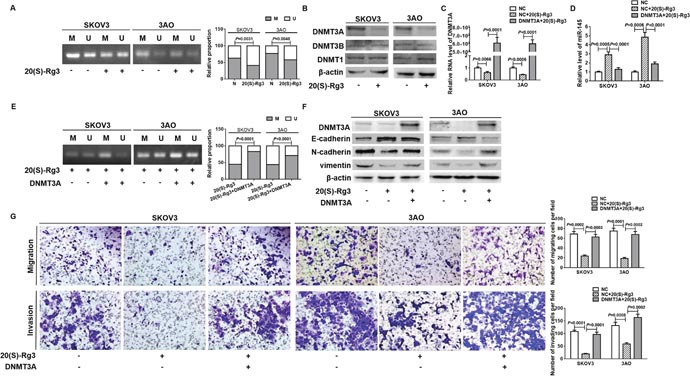
20(S)-Rg3 upregulated miR-145 via suppressing DNMT3A-mediated methylation of pre-miR-145 **(A)** MSP showed that the methylation level in the pre-miR-145 promoter of SKOV3 and 3AO cells was decreased by 20(S)-Rg3. M: methylated product, U: unmethylated product. Fisher exact test. **(B)** Western blot analysis indicated that DNMT3A rather than DNMT3B and DNMT1 was significantly decreased in SKOV3 and 3AO cells treated by 20(S)-Rg3. **(C)** qRT-PCR showed that transfection of DNMT3A plasmid rescued DNMT3A level in 20(S)-Rg3-treated cells. t test. **(D)** qRT-PCR showed overexpression of DNMT3A reversed 20(S)-Rg3-induced miR-145. **(E)** MSP showed the methylation level in the pre-miR-145 promoter was increased in cells treated with both 20(S)-Rg3 and DNMT3A plasmid compared to that in cells treated with 20(S)-Rg3 alone. M: methylated product, U: unmethylated product. Fisher exact test. **(F)** Western blot analysis showed that restoration of DNMT3A reversed 20(S)-Rg3-induced E-cadherin increase, N-cadherin and vimentin decrease. **(G)** *In vitro* migration assay showed that transfection of DNMT3A plasmid stimulated motility and invasion of 20(S)-Rg3-treated cells (200×). t test. All experiments were performed in triplicate and data were showed as means ± SE.

Since DNA hypermethylation has been connected to miR-145 deregulation in prostate cancer [27], we compared the methylation status of miR-145 precursor gene in 20(S)-Rg3-treated cells relative to non-treated cells. The initial assessment of potential CpG islands in the 2000 bp upstream genomic sequence encoding pre-miR-145 found no CpG islands but some CG sites. The MSP results illustrated that the methylation level in the promoter region of pre-miR-145 promoter was decreased in 20(S)-Rg3-treated SKOV3 and 3AO cells (Figure 2A). We thus detected the influence of 20(S)-Rg3 on the expression of DNMT family members. Western blot results showed that DNMT3A, the DNA methyltransferase responsible for de novo methylation, was obviously decreased while DNMT1 and DNMT3B were remained unchanged in 20(S)-Rg3-treated SKOV3 and 3AO cells (Figure 2B). DNMT3A plasmid was transfected into 20(S)-Rg3-treated cells to restore the expression of DNMT3A (Figure 2C), which reversed the 20(S)-Rg3-triggered upregulation of miR-145 (Figure 2D). In parallel, MSP results showed that overexpression of DNMT3A increased the methylation level in the promoter region of pre-miR-145 in 20(S)-Rg3-treated SKOV3 and 3AO cells (Figure 2E). These results indicated that 20(S)-Rg3 enhanced miR-145 via inhibiting DNMT3A expression and thus relieved the methylation restrain on pre-miR-145 transcription.

We next assessed the effects of DNMT3A on the anti-EMT activity of 20(S)-Rg3. DNMT3A overexpression reversed 20(S)-Rg3-triggered E-cadherin upregulation, N-cadherin and vimentin downregulation (Figure 2F). And the attenuation of migration and invasion of 20(S)-Rg3-treated cells was largely reversed by ectopic expression of DNMT3A (Figure 2G). These results showed that 20(S)-Rg3 blocked EMT through downregulating DNMT3A in ovarian cancer cells.

### miR-145 inhibited DNMT3A-promoted EMT

**Figure 3:**
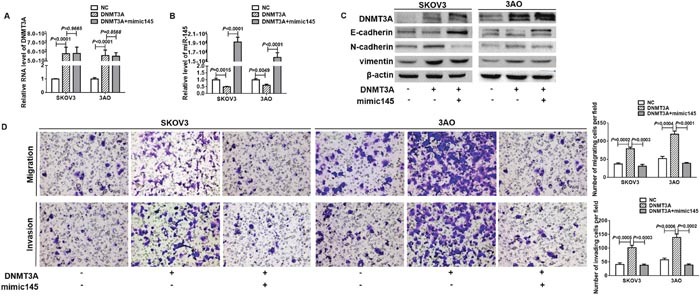
miR-145 blocked DNMT3A-induced EMT **(A)** qRT-PCR showed that ectopic expression of miR-145 had negligible effect on DNMT3A expression in DNMT3A-overexpressed cells. **(B)** qRT-PCR showed that DNMT3A overexpression downregulated miR-145 expression which was recovered by mimic145 transfection. **(C)** Western blot analysis indicated that E-cadherin decrease, N-cadherin and vimentin increase caused by DNMT3A were reversed by concomitant miR-145 overexpression. **(D)** *In vitro* migration and invasion assays indicated that overexpression of miR-145 largely attenuated DNMT3A promotion on the migration and invasion (200×). All experiments were performed in triplicate and datawere showed as means ± SE. t-test.

We further studied the role of miR-145 in DNMT3A-promoted EMT. Ectopic expression of miR-145 in DNMT3A-overexpressed cells (Figure 3A) did not in turn affect DNMT3A expression (Figure 3B), but blunted the EMT-inducible activity of DNMT3A as shown by restoration of E-cadherin, loss of N-cadherin and vimentin (Figure 3C), and attenuation of cell motility and invasion (Figure 3D). These results showed that DNMT3A induced EMT via inhibiting miR-145.

### miR-145 directly targeted FSCNI to inhibit EMT

**Figure 4:**
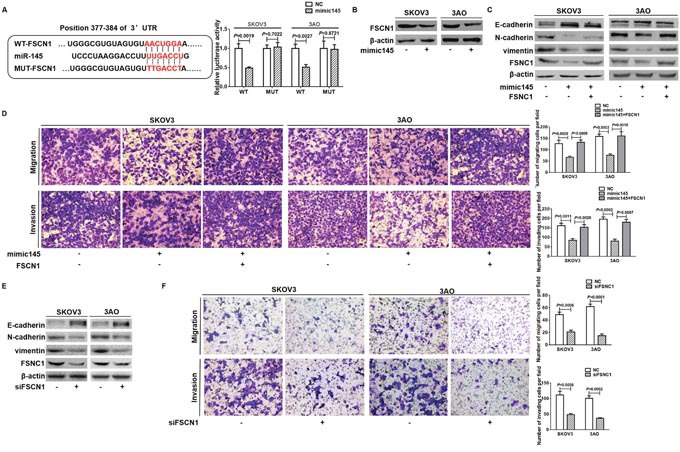
miR-145 directly targeted FSCNI to inhibit EMT **(A)** Schematic representation of FSCN1 WT-3′UTR and MUT-3′UTR sequences used for construction of luciferase report vectors. Luciferase reporter assay showed that, compared to negative control, miR-145 significantly diminished luciferase activity of wild type FSCN1 3′-UTR, while had no effect on luciferase activity of the mutated FSCN1 3′-UTR. **(B)** mimic145-mediated overexpression of miR-145 markedly repressed FSCN1 protein level in SKOV3 and 3AO cells. **(C)** Western blot assays showed that E-cadherin was deceased whereas N-cadherin and vimentin were increased in cells by overexpression of FSCN1 and miR-145 relative to overexpression of miR-145 alone. **(D)** *In vitro* migration and invasion assays showed that FSCN1 overexpression in SKOV3 and 3AO cells largely counteracted miR-145 inhibition on cell migration and invasion (200 ×). **(E)** Western blot assays showed that knocking down of FSCN1 caused the E-cadherin upregulation, N-cadherin and vimentin downregulation. **(F)** *In vitro* migration and invasion assays showed that FSCN1 inhibition in SKOV3 and 3AO cells mitigated cell motile and invading capacity (200 ×). All of the treatments were carried out in triplicate, and the results are displayed as the means ± SE. t-test.

Next we explored the target of miR-145 to block EMT. We first predicted putative target genes of miR-145 by searching the TargetScan database (release 5.1, http://www.targetscan.org/), and FSCN1 was chosen to be experimentally verified. 3′-UTRs of FSCN1 containing the wild-type or mutant putative miR-145 binding site were cloned into a luciferase reporter plasmid, respectively. Specifically, although 4 conserved miR-145 seeding sequences were predicted to exist in the 3′-UTR of FSCN1, only one of them was experimentally confirmed as an actual binding site which was cloned into a luciferase reporter plasmid in our study [28]. The luciferase reporter assay showed that luciferase activity was significantly inhibited in cells co-transfected with miR-145 mimic and FSCN1 WT-3′ UTR vector, while no changes of luciferase activity were detected in cells transfected with miR-145 mimic and luciferase reporter plasmids containing the mutant seeding sequence (Figure 4A). Additionally, western blot analysis showed that miR-145 overexpression diminished FSCN1 expression level (Figure 4B). These data indicated the direct suppressive effect of miR-145 on FSCN1. Since the role of FSCN1 in EMT was not fully explored, we investigated whether FSCN1 mediated the inhibitory effect of miR-145 on EMT. In cells overexpressed both FSCN1 and miR-145, the anti-EMT effect of miR-145 as evidenced by upregulation of E-cadherin, downregulation of N-cadherin and vimentin (Figure 4C), and attenuation of migration and invasion (Figure 4D) was largely reversed by ectopic expression of FSCN1. Meanwhile, EMT was reversed in SKOV3 and 3AO cells by downregulation of FSCN1, as shown by increased expression of E-cadherin, decreased expression of N-cadherin and vimentin (Figure 4E), and demotion of migration and invasion (Figure 4F). Taken together, our data suggested that miR-145 blocked EMT by targeting FSCN1 in ovarian cancer cells.

### miR-145 inhibited ovarian cancer EMT *in vivo*

**Figure 5:**
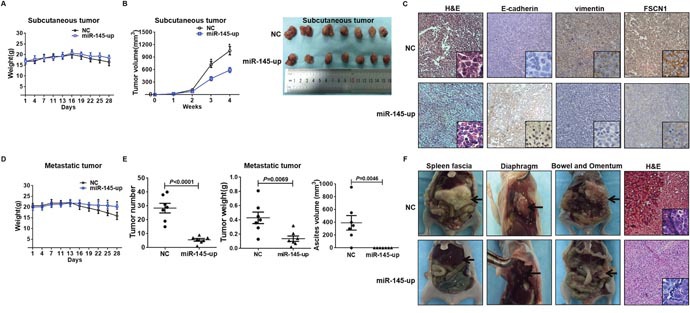
miR-145 inhibited ovarian cancer EMT *in vivo* **(A)** Cells were injected subcutaneously into the nude mice flank. The body weights of nude mice with subcutaneous xenograft were monitored every other day. No differences in body weight were observed between NC and miR-145-up groups. Values are presented as the means (g) ± SD (n= 7 per groups). **(B)** The volumes of subcutaneous tumors were calculated for 4 weeks, which showed that miR-145 strongly inhibited tumor growth in nude mice. Data represents the mean (mm3) ± SD (n= 7 per groups). **(C)** Immunohistochemical staining of E-cadherin, vimentin and FSCN1 expression in subcutaneous tumor samples showed that E-cadherin was increased while vimentin and FSCN1 were decreased in miR-145 up tumors relative to that in NC group (original magnification, 100×; insets, 400×). **(D)** Cells were inoculated into the abdomen of nude mice. The body weights of nude mice with metastatic tumors were monitored every other day. Mice in miR-145-up group lost less body weight than NC group. **(E)** The overall number of metastases, tumor weight, and the volume of ascites were measured. Data are presented as the means (g) ± SD (n = 7 per groups). **(F)** The representative images of xenografts of spleen fascia, diaphragm, bowel and omentum, and the H&E staining of the tumor samples. The xenografts in miR-145-up group were obviously smaller in size than those in NC group.

To determine the effect of miR-145 on ovarian cancer progression *in vivo*, the primary tumor growth in nude mice were examined. miR-145-up SKOV3 cells (transfected by miR-145-expressing lentivirus) or negative control cells (NC) were subcutaneously inoculated into nude mice. All of the mice developed subcutaneous xenografts. Although no differences in body weight were detected between NC and miR-145-up groups (Figure 5A), the average tumor volume of miR-145-up group was reduced by nearly 40% at week 4 compared with the NC group (Figure 5B). Immunohistochemistry analysis of subcutaneous tumors showed that E-cadherin was higher, vimentin and FSCN1 were lower in miR-145-up tumors compared to NC tumors (Figure 5C).

We then examined the inhibitory effect of miR-145 on the intraperitoneal dissemination of ovarian cancer. NC or miR-145-up SKOV3 cells were inoculated into the abdomen of nude mice. During the 28-day observation, the average body weigh was higher in miR-145-up group than NC group, which became significant since day 22 (Figure 5D). And less metastatic tumor nodules and ascites were developed in miR-145-up group (Figure 5E). Intraperitoneal tumor nodules presented throughout the mice’s abdominal cavity. Tumors in spleen fascia and diaphragm of miR-145-up mice were substantially smaller in size than those in NC mice, and immunohistochemistry analysis of metastatic tumor showed the tumors were from the intraperitoneal dissemination (Figure 5F). These data reproduced the suppressive action of miR-145 on ovarian cancer cell *in vivo*.

## DISCUSSION

**Figure 6:**
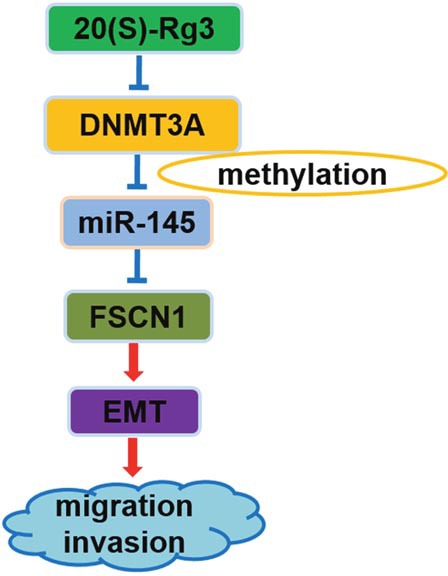
Schematic representation of the anti-EMT mechanism of 20(S)-Rg3 20(S)-Rg3 down-regulated DNMT3A to demethylate pre-miR-145 and thus upregulated mature miR-145 that targeted FSCN1 and finally blocked EMT to attenuate cell migration and invasion.

20(S)-Rg3 inhibited cancer cell motility and invasiveness, and the mechanism could be partly attributed to its anti-EMT effect [29–31]. Here, we first reported that the pathway composed of DNMT3A, miR-145, and FSCN1 was implicated in the anti-EMT mechanism of 20(S)-Rg3 (Figure 6), and that the methylation repression of miR-145 by DNMT3A played an important role in EMT occurrence.

Ginseng, is one of the oldest herbal medicines and induces a variety of physiological and pharmacological effects. Ginseng contains saponins called ginsenosides, which are considered as the biologically active ingredients in ginseng. Many researches have proposed that ginsenoside blocks EMT in cancers. Zhang and colleagues have recently shown that ginsenoside 25-OCH3-PPD, isolated from Panax notoginseng and belonged to protopanaxadiols group as 20(S)-Rg3, reduces EMT markers in normoxically cultured breast cancer cells [32]. Xie et al. have reported that ginsenoside Rg1, a major active component also isolated from Panax notoginseng but belonged to protopanaxatriol group, blocks TGFβ1-induced EMT in rat renal tubular epithelial cells [33]. Collectively, these findings, together with our data, shed new light on the anti-metastasis mechanism of ginsenosides. Nevertheless, the pathway relative to the entrance of the ginsenosides into cells and the direct targets of the ginsenosides have been yet to be determined. It is worthwhile to identify the specific mechanism mediating the anti-tumor role of ginsenosides.

DNA methylation is a critical epigenetic signature that is involved in transcriptional regulation, genomic imprinting, and silencing of repetitive DNA elements [34]. Abnormal hypermethylation correlates with the transcriptional repression of multiple miRNAs. This can lead to the upregulation of oncogenic targets of microRNAs and constitutive activation of signaling pathway that can increase invasion and migration activities [35]. However, to our knowledge, there have been no reports about the methylation regulation of miR-145 in ovarian cancer. In this study, we found that miR-145 was under control of DNMT3A-mediated DNA methylation, and 20(S)-Rg3 inhibited DNMT3A expression to demethylate pre-miR-145 and thus increase miR-145 expression.

DNMT3A together with DNMT3B and DNMT1 are catalytically active DNMTs responsible for genome methylation [36]. DNMT3A and DNMT3B are de novo methyltransferase with different targets [37]. DNMT1 is a maintenance DNA methyltransferase for retaining methylation pattern, with inefficient de novo methylation ability. Increasing evidence shows that these DNMTs work together to maintain a normal methylation pattern, and deregulation of either one could promote malignancies [38]. More recently, DNMT3A has been reported as contributor of EMT [39, 40]. In the present study, we showed that DNMT3A induced EMT via decreasing miR-145 expression.

Decreased expression and anti-tumor function of miR-145 have been observed in several cancers [41, 42]. The mechanistic studies about miR-145 inhibition on cancer progression have initially focused on its roles in cell apoptosis, cell cycle and cell proliferation [43, 44]. Lately, miR-145 has been inversely connected to cancer cell motility and invasiveness, and the mechanism may partly be attributed to its anti-EMT effect [45, 46]. FSCN1 [47] is a newly identified miR-145 target in a few cancers. FSCN1 is an actin-bundling protein functioning in cell movement under physiological or pathological conditions [48]. Overexpression of FSCN1 promotes migration and invasion of cancer cells [7, 49–53], and is associated with clinically unfavorable phenotypes in human epithelial cancers including EOC [48–54]. Nevertheless, the correlation of miR-145-FSCN1 with EMT has not been compellingly proven in the published data. Here we provided the evidence that overexpression of FSCN1 was sufficient to confer EMT to ovarian cancer cells, and its aberrant elevation in ovarian cancer tissues was possibly benefited from miR-145 diminution. We provided evidence that miR-145 inhibited EMT by suppressing FSCN1, however, the detailed mechanism about function of FSCN1 in EMT occurrence still necessitated further elucidation.

In conclusion, we found that 20(S)-Rg3 reversed EMT to inhibit ovarian cancer cells migration and invasion via antagonizing DNMT3A-mediated methylation of pre-miR-145 to promote inhibition of miR-145 on FSCN1. 20(S)-Rg3 is an efficient, low-toxicity and multi-target natural agent, and it is expected to become an important agent in the comprehensive treatment of ovarian cancer, which may improve the current strategy of ovarian cancer therapy.

## MATERIALS AND METHODS

### Reagents and antibodies

Ginsenoside 20(S)-Rg3 was obtained from Tasly Pharmaceutical Company (Tianjin, China) and dissolved at a concentration of 4 mg/ml in DMSO as a stock solution (stored at −20°C). It was then further diluted in cell culture medium to create working concentrations. The maximum final concentration of DMSO was less than 0.1% for each treatment, and was also used as a control. Antibodies including DNMT3A, DNMT3B, E-cadherin, N-cadherin, vimentin, and β-actin were from Cell Signaling Technology (Beverly, MA), FSCN1 was from Abcam (Cambridge, MA, USA), and DNMT1 was from Active Motif (Carlsbad, CA, USA).

### Cell culture and 20(*S*)-Rg3 treatment

The human ovarian cancer cell line SKOV3 was obtained from the Shanghai Cell Bank of Chinese Academy of Sciences (Shanghai, China), 3AO was from the Shandong Academy of Medical Sciences (Jinan, China). Cells were maintained in RPMI 1640 medium (Gibco-BRL, Gaithersburg, MD, USA) supplemented with 10% (v/v) fetal bovine serum at 37°C under a humidified 5% CO_2_ atmosphere. Cells were incubated with 80 μg/ml (for SKOV3) or 160 μg/ml (for 3AO) of 20(S)-Rg3 for 24 h.

### Quantitative real-time PCR (qRT-PCR)

Total RNA was extracted from cells using TRIzol reagent (Invitrogen, Carlsbad, CA, USA) according to the manufacturer’s instructions. Concentration and quality of total RNA were assessed by absorbance at 260 nm and the ratio of 260/280, respectively, on a UV spectrophotometer (BioRad Inc., Hercules, CA, USA). For mRNA detection, first-strand cDNA was synthesized using a RevertAid first strand cDNA synthesis Kit (Thermo Fisher Scientific Inc., Waltham, MA, USA). Quantitative real-time PCR was performed using a SYBR Premix Ex Taq™ II kit (Takara, Dalian, China) on a CFX96 real-time PCR system (Bio- Rad, Hercules, CA, USA). miR-145 was normalized to small nuclear U6, while DNMT3A normalized to β-actin. Relative gene expression was calculated automatically using 2^−ΔΔCt^. Primers for miR-145 and U6 reverse transcription and amplification were designed and synthesized by Ribo-Bio Co., Ltd. (Guangzhou, China). The following primer sequences were used: DNMT3A forward: 5′-TATTGATGAGCGCACAAGAGAGC-3′; DNMT3A reverse: 5′-GGGTGTTCCAGGGTAACATTGAG-3′; β-actin forward: 5′-TCCCTGGAGAAGAGCTACGA-3′; β-actin reverse: 5′-AGCACTGTGTTGGCGTACAG-3′.

### Western blot

Total protein was collected from cells by RIPA lysis buffer containing protease inhibitors (Roche, Indianapolis, IN, USA) and 1 mM PMSF on ice. Protein concentration was measured using the BCA-200 Protein Assay kit (Pierce, Rockford, IL, USA). After heat denaturation at 100°C for 5 min, proteins were separated by electrophoresis on 10% SDS–PAGE gels and then transferred onto nitrocellulose membranes (Pall Life Science, NY, USA). The membranes were blocked with 5% non-fat milk at room temperature for 1 h, and then incubated overnight at 4°C with rabbit anti-human E-cadherin(1:1000, Cell Signaling Technology, Danvers, MA, USA), N-cadherin(1:1000, Cell Signaling Technology, Danvers, MA, USA), vimentin(1:500, Cell Signaling Technology, Danvers, MA, USA), DNMT3A (1:500, Cell Signaling Technology, Danvers, MA, USA), DNMT3B(1:500, Cell Signaling Technology, Danvers, MA, USA), DNMT1(1:1000, Active Motif; Carlsbad, CA, USA), FSCN1(1:100000, Abcam, Cambridge, MA, USA), and mouse anti-human β-actin(1:1000, Cell Signaling Technology, Danvers, MA, USA). After washing with TBST, the blots were incubated with horse radish peroxidase (HRP)-conjugated goat anti-rabbit or anti-mouse IgG. Blots were visualized using ECL reagents (Pierce, Rockford, IL, USA) by a chemiluminescence imaging system (Bio-Rad, Richmond, CA, USA).

### Plasmid transfection

The human FSCN1 expression vector pLenti6/V5-DEST-FASCIN was a gift from Lynda Chin (Addgene, #31207)[55]. The human DNMT3A expression vector pcDNA3/Myc-DNMT3A was a gift from Arthur Riggs (Addgene plasmid # 35521)[56]. SKOV3 and 3AO cells were seeded into 6-well plates until 70%-90% confluency and transiently transfected with pcDNA3/Myc-DNMT3A (pLenti6/V5-DEST-FASCIN) or control vector 3μg per well using the X-treme GENE HP DNA Transfection Reagent (Roche, Indianapolis, IN, USA) following the manufacturer’s protocol. After 48 hours of transfection, the cells were harvested for further study.

### MicroRNA mimic or inhibitor transfection

miR-145 mimic (inhibitor) and negative control were purchased from Ribo-Bio Co. Ltd. (Guangzhou, China). SKOV3 and 3AO cells were seeded into 6-well plates to reach 40%–50% confluency after 24 h and then transiently transfected with 60 nM miR-145 mimic (SKOV3) or 100 nM miR-145 inhibitor or negative control using the X-treme GENE siRNA Transfection Reagent (Roche, Indianapolis, IN, USA). After 24 h of transfection, the cells were treated with 80 μg/ml (SKOV3) and 160 μg/ml (3AO) of 20(S)-Rg3 for 24 h.

### siRNA and transient transfection

Human FSCN1 siRNA was purchased from GenePharma (Shanghai, China). Ovarian cancer cells were seeded into 6-well plates until they reached 40%–50% confluency. FSCN1 siRNA (GCAGCCTGAAGAAGAAGCA) was transiently transfected 100nM per well using the X-treme GENE siRNA Transfection Reagent (Roche, Indianapolis, IN, USA). After 48 hours transfection, the cells were harvested for further studies.

### Cell migration and invasion assay

After treated, cells were trypsinized and counted. A total of 1×10^5^ cells (for migration assay) or 5×10^5^ cells (for invasion assay) in 100 μl serum-free medium were added into millicells (Millipore Co., Bedford, MA, USA) without (for migration assay) or with (for invasion assay) Matrigel (Becton Dickinson Labware, Bedford, MA, USA) coated. 500 μl of 1640 medium containing 20% newborn bovine serum was added to the bottom chambers as the chemotactic factor. After incubation for 24 h (for migration assay) or 48 h (for invasion assay) at 37°C, cells remaining on the upper surface of the filter were removed using cotton swabs. Then the migrated cells were fixed using methyl alcohol and stained using 0.1% crystal violet. Migratory (or invasive) cells were counted and averaged from images of five random fields (original magnification ×200) captured using an inverted light microscope. The mean values of three duplicate assays were used for statistical analysis.

### DNA bisulfite modification and methylation-specific PCR (MSP)

Cells treated by 80 μg/ml (SKOV3) and 160 μg/ml (3AO) for 24 h in 24-well plates were trypsinized and resuspended in cold PBS at the concentration of ∼6×10^6^/ml. DNA bisulfite modification and purification were performed using an EZ DNA methylation-Direct kit (ZYMO RESEARCH, California, USA) according to its instructions. Concentration of DNA was evaluated by absorbance at 260 nm on a UV spectrophotometer (BioRad Inc., Hercules, CA, USA). The set of primers for MSP was flanking the 3kb 5′-region upstream from the start of pre-miR-145 sequence. The primers for methylation-specific PCR were designed by MethPrimer and the sequences were as follows: methylated (M) forward: 5′-GGAGATTGGGGAATATATATGAGTC-3′; methylated (M) reverse: 5′-AAAATAAAATACCACACGTCGC-3′; unmethylated (U)- forward: 5′-AGATTGGGGAATATATATGAGTTGT-3′; unmethylated (U)- reverse: 5′-ACCAAAATAAAATACCACACATCAC-3′. DNA amplification was performed with Epi Taq HS (Takara, Dalian, China) under the following condition: 94°C for 5 min; 30 cycles of 94°C for 30 s, 50°C for 30 s, 72°C for 30 s; and 72°C for 10 min. The PCR productions were separated by 2.0% agarose gel electrophoresis and visualized by a Bio-Rad image lab system (Richmond, CA, USA).

### Luciferase reporter plasmid construction

The wild-type 3′-UTR sequence of the target gene carrying a putative miR-145 binding site was amplified by PCR. To generate mutant 3′-UTR fragment of miR-145 target gene, we adopted the two-step PCR method as reported previously. Briefly, two fragments of 3′-UTR were amplified by two sets of overlapped primers in which mutated seeding sequences of miR-145 were introduced. The wild-type and mutant PCR products were digested with Hind III and Sac I enzymes, inserted into pMIR-REPORT^™^ Luciferase vectors (Ambion, Austin, TX, USA) and verified by DNA sequencing. The following primers were used: FSCN1-WT 3′UTR forward: 5′-CCTCGCTCTGGGAGTACTAGGG-3′; FSCN1-WT 3′UTR reverse: 5′ -CTGGGGCTGCAGACTGAGTTAT-3′; FSCN1-MUT 3′UTR P1: 5′-GCGGCTCGAGCCTCGCTCTGGGAGTACTAGGG-3′; P2: 5′-AATGCGGCCGCCTGGGGCTGCAGACTGAGTTAT-3′; P3: 5′-CAAAAGATAGGTCAAACACTACACGCCCAGGGC-3′; P4: 5′-TGTAGTGTTTGACCTATCTTTTGCCTCTCC CAG-3′.

### Luciferase reporter assay

Luciferase reporter assays were carried out in both SKOV3 and 3AO cells. Cells were seeded into 24-well plate. When reached 80%-90% confluency, cells were co-transfected with pRL-TK vector (20 ng), wild-type (WT-3′ UTR) or mutant (MUT-3′ UTR) reporter vectors (180 ng), along with miR-145 mimic or negative control at a final concentration of 20 nM using the X-treme GENE siRNA Transfection Reagent. Transfections were performed in triplicate. 24 h after transfection, the relative firefly luciferase activity (normalized to Renilla luciferase activity) was measured using a dual-luciferase reporter gene assay system (Promega, Madison, WI, USA), and results were depicted as the percentage change over the respective control. Each experiment was performed in triplicate.

### Animal study

The care and use of experimental animals were approved by the ethical committee of the First Affiliated Hospital of Xi’an Jiaotong University, and were adherent to the institutional guidelines and ethical standards. SKOV3 cells were infected with miR-145-expressing lentivirus GV209-miR145 (GENECHEM, Shanghai, China) or negative control lentivirus GV209 (GENECHEM, Shanghai, China). For subcutaneous xenograft experiment, cells were trypsinized and resuspended in PBS at a final concentration of 2×10^7^cells/ml. 2×10^6^ cells were injected subcutaneously into the flank of six-week old BALB/c nude mice. Animal body weights and tumor perpendicular diameters were recorded every two days. Tumor volumes were calculated according to the formula of V=0.5236×(L×W^2^)(V: tumor volume, L: length, W: width). The mice were killed after 28 days on the experimental treatments. Tumor samples were fixed in 4% polyformaldehyde for 24h at room temperature, paraffin-embedded, and sectioned for immunohistochemical analysis. For intraperitoneal xenograft model, SKOV3 cells were trypsinized and resuspended in PBS at a concentration of 1×10^8^/ml. Mice were inoculated with 1×10^7^ cells in the abdominal cavity at day 0. The mice were observed daily and sacrificed after 28 days. Necropsies were performed, disseminated lesions were resected from mice and pathologically confirmed using HE staining. Ascitic fluid was collected by 1mL syringe and measured by pipet. The number and weight of tumor nodules on the surface of the spleen fascia, diaphragm, bowel and omentum, liver and bladder were counted, measured and statistically analyzed.

### Immunohistochemistry analysis

Paraffn-embedded xenograft tissue sections on poly-1-lysine-coated slides were deparaffnized and rinsed with 10 mM Tris–HCl (pH 7.4) and 150 mM sodium chloride. Paroxidase was quenched with methanol and 3% hydrogen peroxide. Slides were then placed in 10 mM citrate buffer (pH 6.0) at 100°C for 20 min in a pressurized heating chamber. Detection of antigens was carried out using incubation with the primary antibodies for 2 hours at room temperature, followed by incubation with HRP-labeled secondary antibody (MaxVision^™^ HRP-Polymer anti-Mouse/Rabbit IHC Kit) at room temperature for 30 minutes and color development with DAB. Negative control specimens were incubated in PBS without the primary antibody under the same conditions. Slides were counterstained with hematoxylin, dehydrated in an ascending alcohol series, and mounted for analysis. Digital images were acquired on an Olympus BH-2 microscope (Tokyo, Japan) installed with the DeltaPix Camera and software (Maalov, Denmark).

### Statistical analysis

All experiments were performed at least in triplicate, and each experiment was independently performed at least 3 times. The graphical presentations were performed using GraphPad Prism 5.0. Data were presented as the means±SE and were analyzed using SPSS 22.0 software (Chicago, IL, USA). Statistical differences were tested by Chi-square test or two-tailed t-test, and Fisher exact test was performed to analysis MSP results. Differences were considered significant at P <0.05 (*) or highly significant at P <0.001 (**).

## SUPPLEMENTARY FIGURES


