# Supplementary material for: 20(S)-Rg3 blocked epithelial-mesenchymal transition through DNMT3A/miR-145/FSCN1 in ovarian cancer
Source: Oncotarget. 2017 Jun 15;8(32):53375–86. doi: 10.18632/oncotarget.18482 (PMC5581117; doi:10.18632/oncotarget.18482)
Supplement: Supplementary file 1 [file oncotarget-08-53375-s001.pdf]

20(S)-Rg3 blocked epithelial-mesenchymal transition through DNMT3A/miR-145/FSCN1 in ovarian cancer

SUPPLEMENTARY MATERIALS

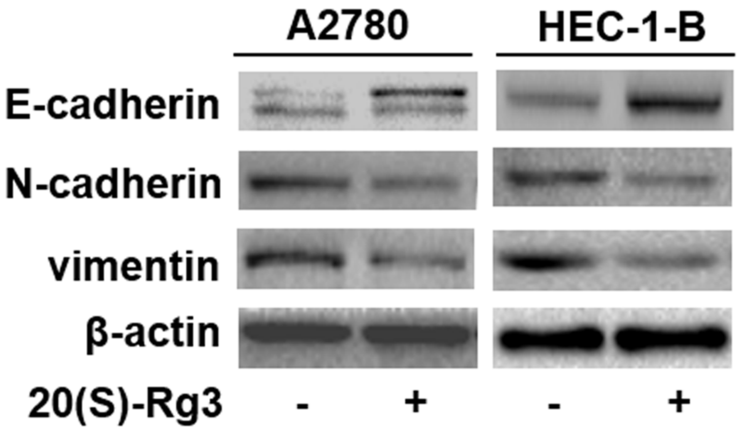

Supplementary Figure 1: 20(S)-Rg3 reversed EMT in A2780 and HEC-1-B cells. Western blot analysis showed that E-cadherin was up-regulated while N-cadherin and vimentin were down-regulated at protein level in 20(S)-Rg3-treated ovarian cancer cell A2780 and endometrial cancer cell HEC-1-B.

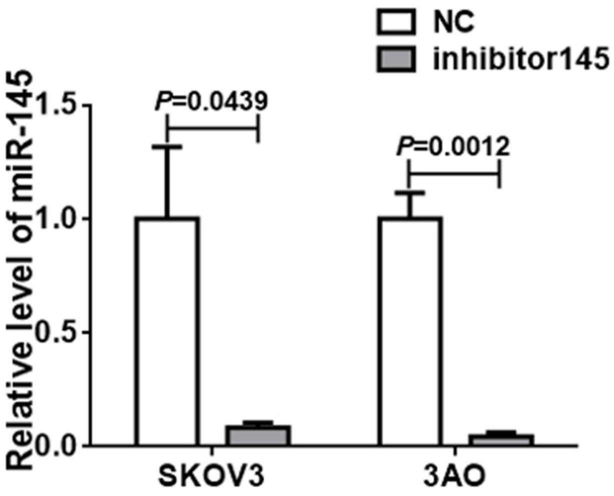

Supplementary Figure 2: Inhibitor145 reduced miR-145 level. qRT-PCR showed that transfection of miR-145 inhibitor downregulated miR-145 level in SKOV3 and 3AO cells. The results were carried out in triplicate, and the results are displayed as the means ± SE. t-test.

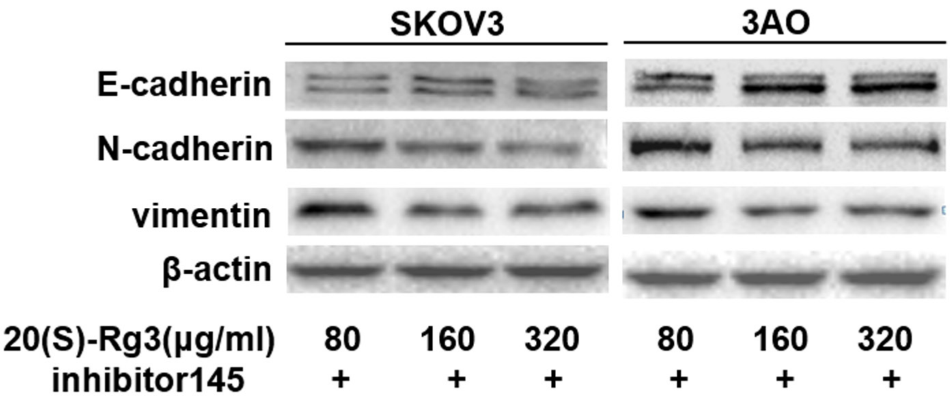

Supplementary Figure 3: 20(S)-Rg3 influenced EMT marker at different concentration. Western blot analysis indicated 160 and 320μg/ml of 20(S)-Rg3 caused slight increase of E-cadherin and decrease of N-cadherin and vimentin relative to that of 80μg/ml 20(S)-Rg3 in ovarian cancer cells.
